# Chemical and Antimicrobial Profiling of Propolis from Different Regions within Libya

**DOI:** 10.1371/journal.pone.0155355

**Published:** 2016-05-19

**Authors:** Weam Siheri, Tong Zhang, Godwin Unekwuojo Ebiloma, Marco Biddau, Nicola Woods, Muattaz Yassein Hussain, Carol J. Clements, James Fearnley, RuAngelie Edrada Ebel, Timothy Paget, Sylke Muller, Katharine C. Carter, Valerie A. Ferro, Harry P. De Koning, David G. Watson

**Affiliations:** 1 University of Strathclyde, Strathclyde Institute of Pharmacy and Biomedical Science, 161 Cathedral Street, Glasgow, G4 0RE, United Kingdom; 2 Wolfson Wohl Cancer Research Centre, Institute of Cancer Sciences, University of Glasgow, Switchback Road, Glasgow, G61 1QH, United Kingdom; 3 Institute of Infection, Immunity and Inflammation, College of Medical, Veterinary and Life Sciences University of Glasgow, Glasgow G12 8TA, United Kingdom; 4 BeeVital, Whitby, North Yorkshire, YO22 5JR, United Kingdom; 5 Dept. of Pharmacy, Health and Well-being, University of Sunderland, Wharncliffe Street, Sunderland SR1 3SD, United Kingdom; University of British Columbia, CANADA

## Abstract

Extracts from twelve samples of propolis collected from different regions of Libya were tested for their activity against *Trypanosoma brucei*, *Leishmania donovani*, *Plasmodium falciparum*, *Crithidia fasciculata* and *Mycobacterium marinum* and the cytotoxicity of the extracts was tested against mammalian cells. All the extracts were active to some degree against all of the protozoa and the mycobacterium, exhibiting a range of EC_50_ values between 1.65 and 53.6 μg/ml. The toxicity against mammalian cell lines was only moderate; the most active extract against the protozoan species, P2, displayed an IC_50_ value of 53.2 μg/ml. The extracts were profiled by using liquid chromatography coupled to high resolution mass spectrometry. The data sets were extracted using m/z Mine and the accurate masses of the features extracted were searched against the Dictionary of Natural Products (DNP). A principal component analysis (PCA) model was constructed which, in combination with hierarchical cluster analysis (HCA), divided the samples into five groups. The outlying groups had different sets of dominant compounds in the extracts, which could be characterised by their elemental composition. Orthogonal partial least squares (OPLS) analysis was used to link the activity of each extract against the different micro-organisms to particular components in the extracts.

## Introduction

Bees collect propolis from plants and use it to coat the inside surfaces of the hive in order to maintain a sterile environment. A wide variety of plant species are used by bees as a source for propolis production, leading to a wide chemical diversity [1]. Even within a fairly limited geographical region such as the UK propolis composition varies substantially [2]. Bees are subject to infection by a range of micro-organisms and these include the protozoal *Crithidia* species, and the *Nosema* species that were originally classified as protozoa but have now been reclassified as fungi. It has been found that *N*. *ceranae* and *N*. *apis* infections are widespread in Scottish beehives [3]. The best-characterised *Crithidia* parasite that infects bees is *Crithidia bombi*, which infects bumble bees [4]. In a recent publication it was found that *Crithidia mellificae* and *Nosema ceranae* infections are associated with winter mortality in European bees [5]. Thus it would seem logical that selection pressure would drive bees to collect phytochemicals that are effective against protozoa and other micro-organisms that could infect the hive [6, 7]. *Crithidia*, which are classified as lower Trypanosomatidae, which are very prevalent in the infection of invertebrates, are closely related to the human pathogens of the genera *Leishmania* and *Trypanosoma* [8]. Since propolis is collected by bees for the specific purpose of providing phytochemical protection against pathogens, there is a strong likelihood of finding highly active antimicrobials in it which might be effective in treating humans [9]. Moreover, the fact that propolis permeates the environment of the beehive makes it likely that it would not be particularly toxic to other multicellular organisms. Libya covers an area of over 1,759,540 km^2^ and the Libyan Desert, which constitutes approximately 90% of Libya, is one of the most arid places on earth. Oases can be found scattered throughout Libya, the most important of which are Ghadames and El-Kufra. The northern regions enjoy a milder Mediterranean climate. Most of the commercial beekeepers are located in an agricultural belt that extends to about 30 km from the coast [10, 11]. Table A in S1 File summarises the main plants in Libya from which bees are known to collect nectar. The current work follows from our earlier work on a sample of propolis collected from the East of Libya, from which four known compounds with activity against *T*. *brucei* and *L*. *donovani* were isolated [12]. The samples studied in this paper represent a larger variety of habitats and climates. The aim of the study was to continue our chemical mapping of the composition of African propolis and carry out anti-parasitic screens in search of high activity samples which might be useful in treating human parasitic infections.

## Materials and Methods

### Materials

Absolute ethanol, HPLC grade acetonitrile, methanol, formic acid and Acrodisc syringe filters were obtained from Fisher Scientific (Loughborough, UK). Chloroform and dimethyl sulphoxide (DMSO) were obtained from Sigma Aldrich, Dorset, UK. HPLC grade Water was produced in-house using a Milli Q system (Millipore, UK).

#### Animals

Age matched inbred BALB/c female mice (20–25 g) in-house bred were used in studies at Strathclyde University. Animal studies were carried out with local ethical approval and had UK Home Office approval (Project license PPL 60/4334).

### Propolis samples

**Fig 1:**
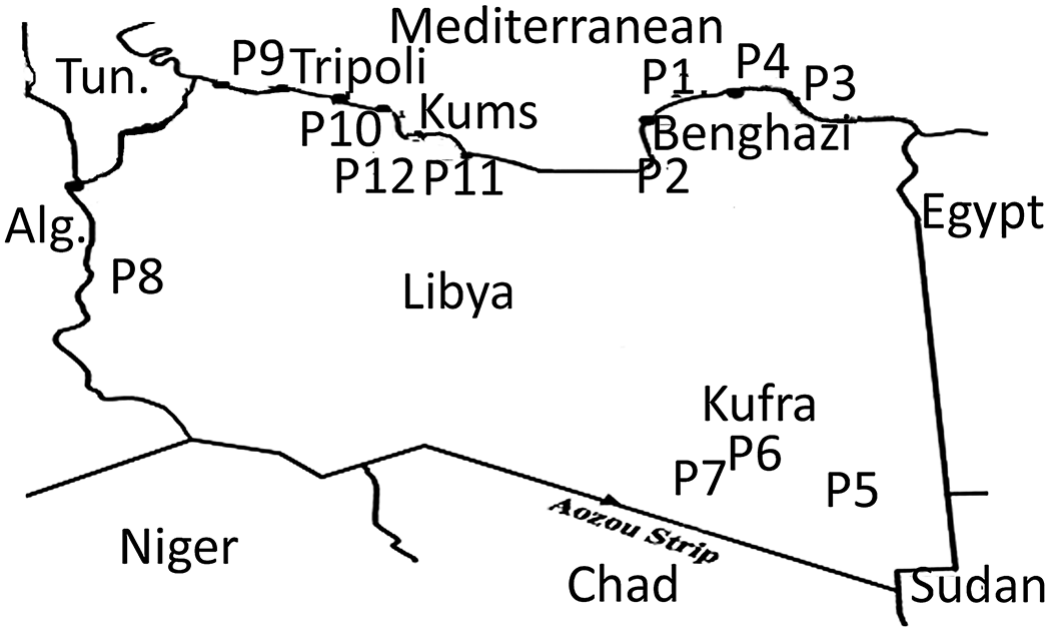
Libyan map showing the collection points Libyan Propolis samples P1 (Alagoria), P2 (Gaminis), P3 (Byda), P4 (Quba), P5,P6, P7 (Kufra), P8(Ghadames), P9 (Tripoli), P10 (Khasr Khiar), P11, P12 (Khumas).

Twelve propolis samples were collected from different Libyan localities: **Tukra** (Al`Aquriyah,) 70 km East of Benghazi (32° 31’ N, 20° 34’ E) (P1); **Qaminis** 53km South of Benghazi (31° 39’ N, 20° 00’ E) (P2); **Bayda** East of Benghazi (32° 45’ N, 21° 44’ E) (P3); **Quba** East of Benghazi (32° 46’ N, 22° 15’ E) (P4); three samples from **Kufra** in South East Libya (24° 15’ N, 23° 18’ E) (P5, P6 and P7); **Ghadames** South West Libya (30° 8’ N, 9° 30’ E) (P8); **Tripoli** North West Libya (32° 54’ N, 13° 11’ E) (P9); **Khaser Khiar** 80 km East of Tripoli (32° 45’ N, 13° 43’ E) (P10) and two samples from **Khumas** 120 km East of Tripoli (32° 38’ N, 14° 15’ E) (P11, P12) (Fig 1). The samples were collected between December 2012 and March 2014. The physical properties of the samples are summarised in Table B in S1 File. The samples were collected by scraping the propolis sample off the top of the hive using a spatula and collecting in a clean tray.

### Sample Extraction

A sample of approximately 20 g of each propolis sample was extracted by sonication in 100 mL of absolute ethanol for 60 min (Clifton ultrasonic bath, Fisher Scientific, Loughborough, UK), after which the extract was filtered and re-extracted twice more with 100 mL of ethanol, filtering each time (Whatman grade 1 filter paper, Fisher Scientific, Loughborough, UK). The extracts were combined, and the solvent was evaporated using a rotary evaporator (Buchi, VWR, Leicestershire, UK), and the residue weighed.

### Anti-parasitic Assays

#### Anti-trypanosomal assay

Testing was carried out against a standard drug-sensitive *T*. *brucei* clone, Lister strain 427 (s427) [13, 14], and the results were expressed as EC_50_ values based on three replicates at each concentration. The assay is based on viable cells metabolizing the blue non-fluorescent dye resazurin to resorufin, which is pink and fluorescent. The assays were performed using serial dilutions in white opaque plastic 96-well plates (F Cell Star, Greiner Bio-one GmbH, Frickenhausen, Germany), with each compound or mixture double diluted over 2 rows of the plate (i.e. 23 double dilutions and a no-drug control well), facilitating an optimally-defined EC_50_ value after plotting of the reading to a sigmoid curve with variable slope (GraphPad Prism 5.0). The seeding density at the start of the assay was 2×10^4^ cells/well, and the cells were exposed for 48 h to the test compounds, at 37°C/5% CO_2_, before the addition of the resazurin dye and a further incubation of 24 h under the same conditions. Fluorescence was determined in a FLUOstar Optima (BMG Labtech, Ortenberg, Germany) at wavelengths of 544 nm and 620 nm for excitation and emission, respectively.

#### Anti-leishmanial assay

Intraperitoneal macrophages were recovered from the peritoneal cavity of BALB/c mice 3 days after intraperitoneal injection with 1mL 3% w/v aqueous sterile starch solution. The mice were then euthanized, and 3mL of incomplete medium (RPMI-1640, 100 μg/mL penicillin–streptomycin and 200 mM L-glutamine) was injected into the peritoneal cavity. The macrophage-containing medium was then removed and collected, and the resulting cell suspension centrifuged at 3000 × g for 5 min and then re-suspended in 10mL complete medium (in complete RPMI-1640 supplemented with 10% heat inactivated fetal calf serum (FCS) [v/v]). The cells were then used in antileishmanial assays. Bone marrow was then harvested from the femurs of each mouse by flushing out the removed bone with 5ml of bone marrow medium (Dulbecco’s modified Eagle’s medium, 20% heat-inactivated fetal calf serum (FCS) [v/v], 30% L-Cell solution [v/v], 100μg/mL penicillin–streptomycin and 200mM L-glutamine). The cell suspension was added to sterile petri dishes (one petri dish/mouse) and incubated for 7 days at 37°C in an atmosphere of 5% CO2:95%air. The medium was removed from the plate, and 7mL TrypLE Express was added to detach the bone marrow-derived macrophages. The resulting suspension of bone marrow-derived macrophages was collected, pelleted by centrifugation and re-suspended in 10mL of incomplete medium and then used in anti-leishmanial assays. The number of live macrophages per millilitre was determined microscopically using a haemocytometer, by mixing a cell sample with 1:1 trypan blue (20 μL) and viewing at ×10 magnification. In all cases, cell viability was >95%. Cells (0.5 × 10^5^ in 200 μL complete medium) were added to the appropriate wells of a 96- well tissue culture plate and incubated for 24 h at 37°C in an atmosphere of 5% CO2:95% air. Cells were then infected with *L*. *donovani* luciferase-expressing promastigotes, produced at the University of Strathclyde using strain MHOM/ET/67:LV82, using a 20:1 parasite/host cell ratio. The plate was incubated as before for 24 h. The medium was removed from each well and replaced with 200 μL complete medium (control, n = 6) or various concentrations of the one of the extracts (diluted in 4% DSMO v/v in complete medium, n = 3) or amphotericin B solution (4–0.02 μg/mL). The plate was incubated as before for 72 h, the medium was then removed, and 150 μL of luciferin solution (150 μg/mL luciferin in complete RPMI-1640) was added to each well. The bioluminescence intensity (BLI) emitted per well was determined using the IVIS^®^ imaging system (Caliper Life Sciences, Runcorn, UK) [12, 15]. The suppression in bioluminescent signal for each test sample was compared with the mean control value. The mean IC50 value was then calculated for each sample by Probit analysis. Data were analysed using MINITAB^®^ software version 16.1.1 supplied by Minitab Ltd. Coventry, UK, and an Anderson–Darling test was used to establish if the data were normally distributed. Parametric data were analysed using a Student’s unpaired t-test or by one-way analysis of variance dependent on the number of treatments/experiments, and significance was confirmed by a Fisher test. A Mann–Whitney or Kruskal– Wallis test was used to analyse data that did not have a normal distribution. Results were considered statistically significant at a p-value of <0.05.

#### Anti-Mycobacterium marinum assay

The anti-bacterial bioassays against *Mycobacterium marinum* (ATCC.BAA535) were performed in 96-well microtitre plates using a modification of the well-established Alamar Blue^™^ method [16, 17]. *M*. *marinum* was inoculated on to a Columbia agar with chocolated horse blood slope (Fisher Scientific, UK) and incubated at 31°C for 5 days. A loopful of the 5 day old *M*.*marinum* culture was transferred to a sterile universal container containing 10 ml saline plus (425–600μm) glass beads (Sigma Aldrich, Dorset, UK). The bacterial suspension was mixed vigorously and allowed to settle, an aliquot of the bacterial suspension was transferred to a tube containing saline, and the turbidity was matched to that of a 0.5 McFarland standard (1.5x10^8^ CFUs/ml) and then diluted with MHB (Cation Adjusted Mueller Hinton Broth, TREK Diagnostic Systems Ltd. UK) to 1.5x 10^7^ CFUs/ml and then 1:1 in the assay microplate to give a final concentration of 0.75 x 10^7^ CFUs/ml. The assay microplate was incubated at 31°C for 6 days, after which 10% Alamar Blue^™^ was added and the incubation continued for a further 24 h. Fluorescence was determined using a Wallac Victor 2 microplate reader (Excitation 560nm Emission 590nm) (Perkin Elmer, Waltham MA, USA). The samples were tested in duplicate over a concentration range of 100–0.19μg/ml and negative and positive controls were included containing 1–0.0019% DMSO and 100–0.78 μg/ml gentamycin respectively

#### Anti-Plasmodium falciparum assays

Activity against *P*. *falciparum* (3D7, The Netherlands) was determined as described previously [18, 19]. Synchronous ring stage parasites were seeded and incubated in triplicate into 96 well plates at 0.5% parasitemia and 2.5% haematocrit, using hypoxanthine free RPMI 1640 (Sigma Aldrich, Dorset, UK) medium, containing 0.5% [v/v] AlbuMAX II (Life technologies, Paisley, UK), 2 mM L-glutamine (Sigma Aldrich, Dorset, UK) and increasing concentrations of each compound (0.1 to 200 μg/mL and no drug control; final DMSO concentration < 0.5% v/v). Increasing concentrations of chloroquine (Sigma Aldrich, Dorset, UK) were used as a positive control (0.05 to 100 nM and no drug control). Parasites were cultured for 48 h before 5 μCi/mL [^3^H]-hypoxanthine (American Radiolabeled Chemicals, Saint Louis MO, USA) was added to each well to be then incubated for an additional 24 h before being frozen at -20°C. After thawing, plates were harvested onto filter mats with a Harvester 96^™^ Mach III (TomTec, Hamden CT, USA) and [^3^H]-hypoxanthine incorporation determined by scintillation counting using a Wallac 1450 MicroBeta Trilux counter (Perkin Elmer, Waltham MA, USA).

#### Anti-Crithidia fasciculata assays

*C*. *fasciculata* (ATCC50083) was grown in RPMI 1640 medium supplemented with L-glutamine and 10% v/v heat inactivated foetal bovine serum for 24 h with shaking prior to use [20]. These cells were then used to inoculate wells of a 96 well plate with 1 x 10^5^ cells per well in 100μl of medium. Stock extracts were prepared in DMSO for each concentration so that there was a constant percentage of DMSO per well (2.5% v/v). The absorbance of plates was determined at 620nm (T_0_) using a Bio Rad xMark Microplate Spectrophotometer (Hemel Hempstead, UK) and plates and these were then incubated for 48 h at 25°C. The absorbance of the wells was then determined again at 620nm (T_48_). For compounds showing no change in absorbance (T_48_-T_0_) terminal subculture was performed and growth determined by absorbance @620nm and by microscopy. Pentamidine was included as a control drug in all assays but it shows variable activity against *C*. *fasciculata* [21] and thus menadione was used as an additional control drug.

### Cell Toxicity Assay

The U937 cells (from the European Collection of Cell Cultures Cat. No. 85011440, supplied by Sigma-Aldrich, Dorset, UK).were grown until approximately 70–80% confluence before plating at 1x10^5^cells/ml in a 96 well plate. The cell plates were then incubated overnight at 37°C, 5% CO_2_. Samples were prepared on a dilution plate in normal cell culture media respective to the cell line used. For initial testing, samples were added to the cells at a range of different concentrations in order to determine the IC50 value for each sample. Samples were serially diluted 1 in 2 from 200μg/ml to 1.56μg/ml. Following the addition of the extracts, the cell plates were incubated for 24 h at 37°C and then resazurin solution was added to a final concentration of 10% (v/v). The cell plates were incubated at 37°C in the dark for 4 h and 24 h before the fluorescence reading (560nm excitation, 590nm emission) was recorded on a Spectramax Plate Reader (Molecular Devices, CA, USA). Each sample was tested in triplicate and the results are expressed as cell viability as a percentage of the cell only control.

### Liquid Chromatography High Resolution Mass Spectroscopy LC-HRMS

A sample of the ethanolic extract of each crude sample (1 mg), was dissolved in methanol (1 mL) and analysed by LC–MS. The separation was performed on an ACE C_18_ column (150 × 3mm, 3 μm) from HiChrom, Reading, UK with 0.1% v/v formic acid in water as mobile phase A and 0.1% (v/v) formic acid in acetonitrile as mobile phase B, at a flow rate of 0.300 mL/min using a gradient as follows: 0–15 min linear gradient from 30% to 50% of B, 15–25 min 50% of B, 25–40 min linear gradient from 50% to 80% of B, 40–50 min 80% of B, 50–51 min increasing to 100% B, 51–59 min at 100% of B with the flow rate increasing to 500 μl/min, 60–70 min 30% of B. The Accela HPLC system was interfaced to and Orbitrap Exactive mass spectrometer (Thermo Fisher, Hemel Hempstead, UK) used in ESI positive negative ion switching mode with needle voltages of +4.5kV and -4.0kV in positive and negative modes respectively. Sheath and drying gas flows were set at 50 and 17 arbitrary units respectively the heated capillary temperature was 275°C. In addition, data dependent MS^n^ fragmentation [19] was carried out by using collision induced dissociation (CID) at 35 V on a LTQ-Orbitrap mass spectrometer combined with a Surveyor HPLC system using the chromatographic method outlined above.

### Software and Data processing

MZMine 2.10 [22] was used for LC-HRMS data processing. The procedure and the settings were the same as described in our previous study [23]. The generated peak lists from both ESI positive and negative modes were imported separately into SIMCA-P 14 (Umetrics, Sweden) for Principal Component Analysis (PCA). The data was Pareto scaled and log transformed prior to PCA modelling. The first 500 LC-HRMS features from each sample were selected based on the mean peak area and putatively identified by searching for the accurate masses against the Dictionary of Natural Products (DNP 2013 version) [24]. The raw data files are publically accessible at: https://pure.strath.ac.uk/portal/en/datasets/search.html with a DOI: 10.15129/0b549ed7-de92-4389-8fa0-a36549a3553b.

## Results

### Propolis Samples Cluster Partly According to Geographic Origin

**Fig 2:**
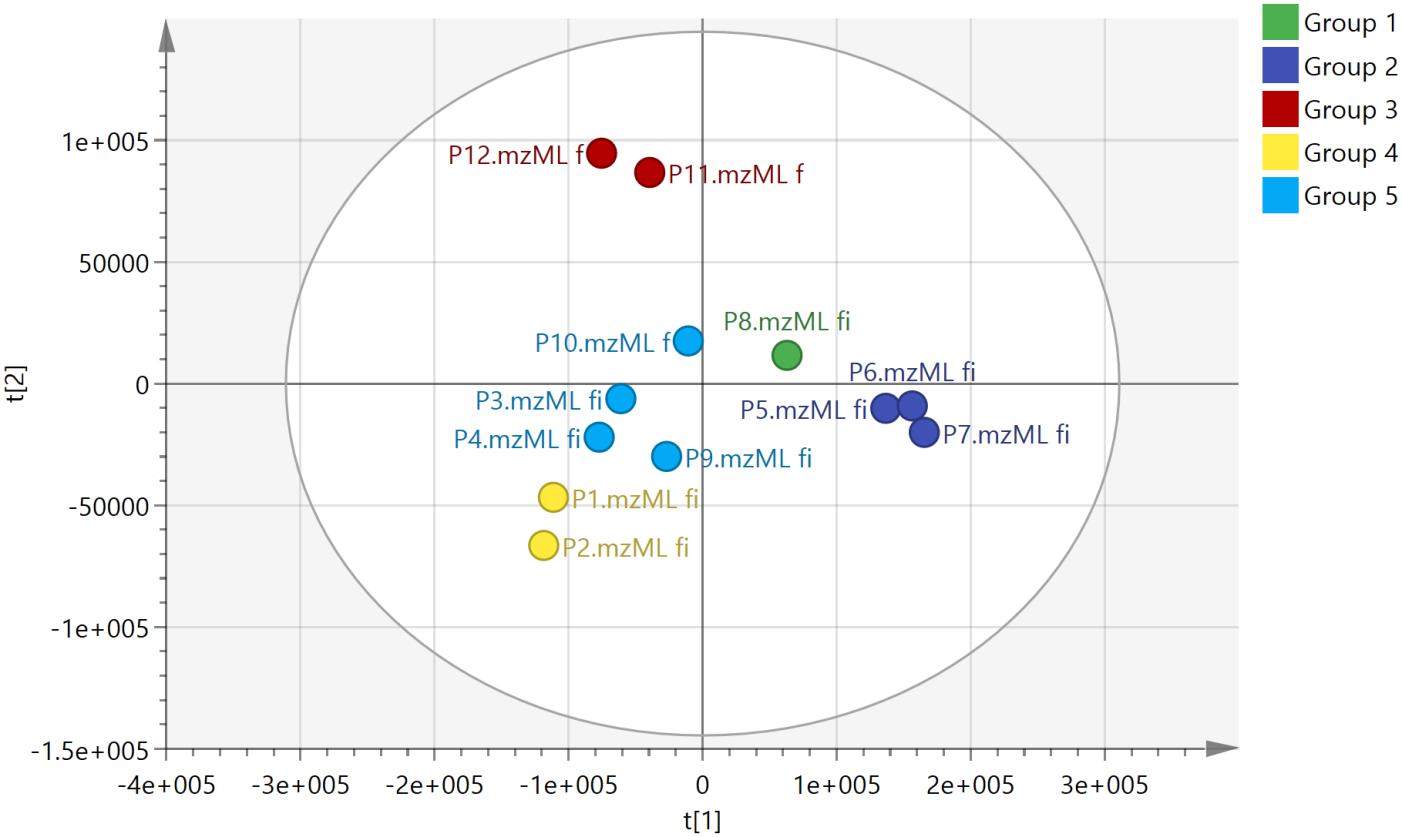
PCA with HCA based on the 300 most intense features obtained in negative ion mode for the 12 propolis samples R^2^X 0.689, Q^2^ 0.48.

In order to get an overview of the differences in the chemical composition of the different propolis samples PCA was used. This method reduces the hundreds of variables (chemical compounds) in the samples to two principle components using the covariance within the data, essentially mapping the samples according to how close they are in composition. Fig 2 shows a PCA based on the 300 features with the highest mean peak areas across the 12 samples selected by m/z mine from the negative ion data which included 30020 features. The R^2^X score for the data was 0.689 indicating that 68.9% of the variation in the data was explained by the first two principal components. Hierarchical cluster analysis (HCA) was used to divide the samples into 5 groups. Only samples P5, P6 and P7 from the SE of the country gave a distinct group and they were grouped close to the sample from the SW (P8). The samples from the coast did not divide according to longitude and the two groups P3, P4, P9, P10 and P11, P12 are composed of samples from the E and W. Although P10 was collected from a site close to P11 and P12 it seems to be quite different in composition. Table 1 lists the ten most important variables (VIPs) used in the PCA classification of the samples for each group [25]. Samples P1 and P2 were similar in composition and three diterpenes and a lignan were previously isolated from sample P2 in our earlier study [12]. However, in the PCA model shown in Fig 2 the most important variables for the classification of the samples are not the diterpenes isolated previously but unknown compounds with m/z values in negative ion mode at m/z 325.145 and m/z 341.140. All masses deviated by < 2 ppm from the proposed elemental composition but, as can be seen in Table 1 the DNP often has many isomeric possibilities matching the elemental compositions of the VIPS. A compound with m/z 373.27 in negative ion mode has the highest importance for locating P5, P6 and P7 and is present in smaller amounts in the other samples. Samples P11 and P12 from the West also have clear marker compounds whereas the weightings of the VIPs in samples P3, P4, P9 and P10 are weak, indicating that these samples have an average composition. Data extraction of the positive ion data yielded 6363 features of which the top 500 by mean intensity were selected for PCA. The groupings obtained were similar to those obtained with the negative ion data (Fig A in S1 File).

**Table 1:** The top 10 VIPs, composed of negative ion masses measured to within 2 ppm of that of the proposed elemental compositions responsible locating the groups shown in Fig 2.

m/z	Rt (min)	Molecular formula	Isomers in DNP	VIP
P1/P2				
325.145	24.925.0	C_20_H_22_O_4_	109	10.1
341.140	21.4	C_20_H_22_O_5_	188	8.2
595.168	3.3	C_27_H_32_O_15_	52	3.5
329.067	11.1	C_17_H_14_O_7_	163	3.5
325.145	10.1	C_20_H_22_O_4_	109	2.8
331.155	17.7	C_19_H_24_O_5_	106	2.7
341.140	13.6	C_20_H_22_O_5_	188	2.6
341.103	10.5	C_19_H_18_O_6_	127	2.5
421.093	14.2	C_23_H_18_O_8_	16	2.4
357.135	29.0	C_20_H_22_O_6_	236	2.2
301.217	43.6	C_20_H_30_O_2_	598	2.0
381.192	8.2	C_20_H_30_O_7_	184	2.0
P5/P6/P7				
373.275	52.6	C_24_H_38_O_3_	45	13.0
401.306	56.4	C_26_H_42_O_3_	27	10.1
375.291	57.4	C_24_H_40_O_3_	27	9.3
369.244	48.8	C_24_H_34_O_3_	11	7.1
385.239	36.8	C_24_H_34_O_4_	45	5.7
345.244	50.0	C_22_H_34_O_3_	127	5.0
387.254	49.1	C_24_H_36_O_4_	51	4.8
347.259	52.9	C_22_H_36_O_3_	114	4.6
361.275	54.9	C_23_H_38_O_3_	24	4.2
371.260	50.3	C_24_H_36_O_3_	21	3.6
P11/P12				
289.108	10.6	C_16_H_18_O_5_	81	13.5
333.171	7.4	C_19_H_26_O_5_	94	12.7
247.098	6.0	C_14_H_16_O_4_	108	8.6
333.171	8.1	C_19_H_26_O_5_	81	8.2
587.339	32.4	C_37_H_48_O_6_	3	7.7
645.308	19.5	C_38_H_46_O_9_	8	7.7
373.166	15.3	C_21_H_26_O_6_	107	7.7
331.155	8.6	C_19_H_24_O_5_	93	7.2
313.145	15.2	C_19_H_22_O_4_	117	6.4
349.166	6.6	C_19_H_26_O_6_	102	6.1
P3/P4/P9/P10				
619.438	47.9	C_40_H_60_O_5_	1	1.5
347.187	19.5	C_20_H_28_O_5_	531	1.2
763.551	57.9	C_48_H_76_O_7_	1	1.0
707.474	9.1	C_40_H_68_O_10_	5	0.9
763.551	53.6	C_48_H_76_O_7_	1	0.8
369.301	47.9	C_22_H_42_O_4_	8	0.7
397.223	12.4	C_21_H_34_O_7_	26	0.7
333.207	14.0	C_20_H_30_O_4_	776	0.6
379.213	20.0	C_21_H_32_O_6_	52	0.6
187.098	6.0	C_9_H_16_O_4_	31	0.5
P8				
401.306	56.4	C_26_H_42_O_3_	27	4.2
345.244	50.0	C_22_H_34_O_3_	127	4.2
371.26	50.3	C_24_H_36_O_3_	21	4.1
375.291	57.4	C_24_H_40_O_3_	27	3.7
369.244	48.8	C_24_H_34_O_3_	11	3.4
255.066	15.6	C_15_H_12_O_4_	145	3.2
347.259	52.9	C_22_H_36_O_3_	114	3.1
373.275	52.6	C_24_H_38_O_3_	45	2.9
375.291	55.6	C_24_H_40_O_3_	27	2.6
397.275	50.8	C_26_H_38_O_3_	23	2.1

The twelve propolis sample extracts were tested for their activity against *P*. *falciparum*, *T*. *brucei*, *L*. *donovani*, *C*. *fasciculata* and *M*. *marinum*. In addition cellular toxicity assays were carried out using mammalian cells.

### Anti-parasitic Activity

#### Activity of propolis extracts against *P*. *falciparum*

**Fig 3:**
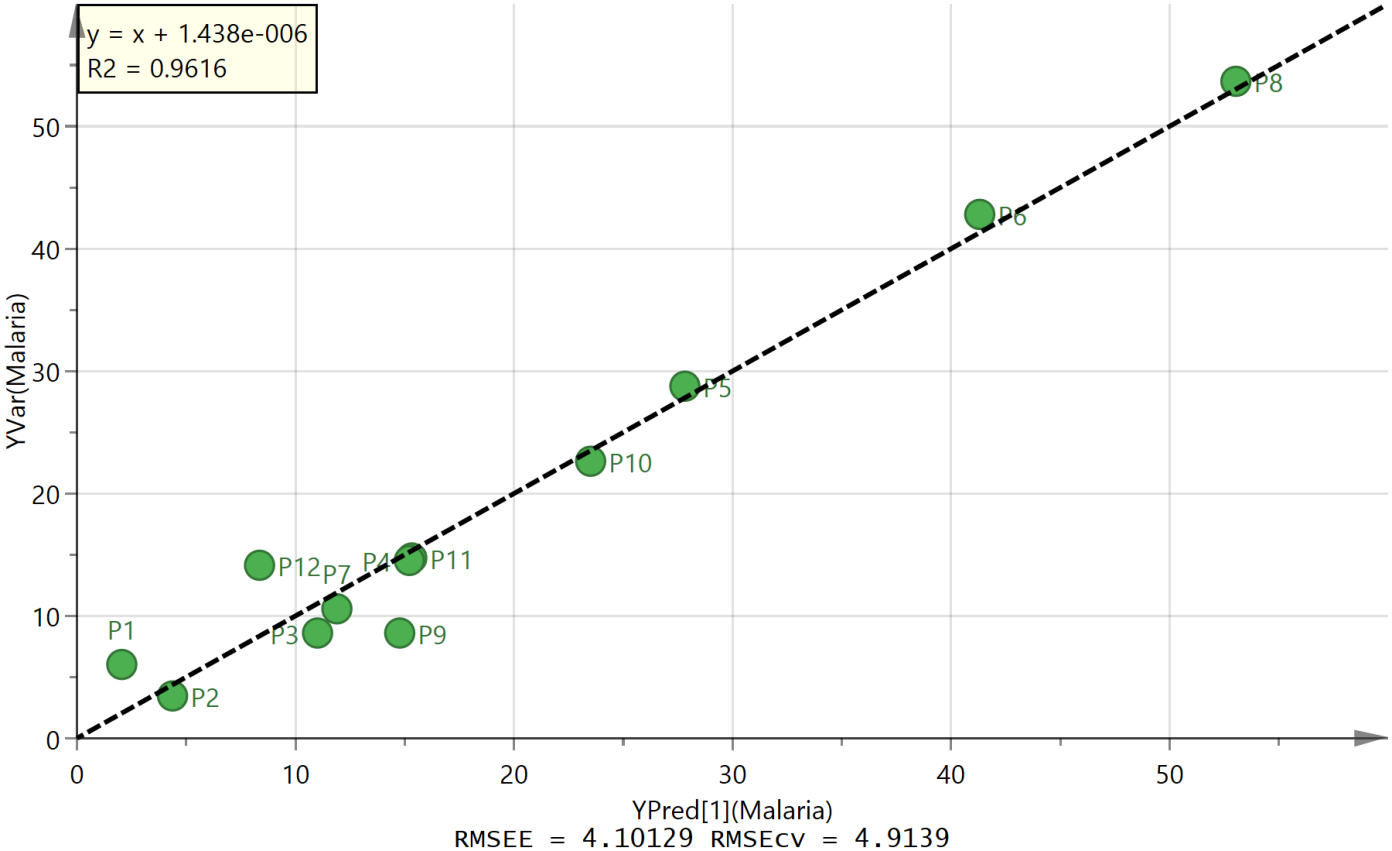
OPLS plot of observed against predicted activity against *P*. *falciparum* based on five compounds (A-E).

Fig 3 shows an OPLS plot for the observed activity of the extracts against *P*. *falciparum* shown in Table 2 constructed using 5 of the 300 variables used to produce Fig 1 by systematically discarding the variables with less impact on the model. The correlation between observed and predicted activity is very good with all the samples falling on the line. Table 3 shows the five most important variables contributing to the high activity of sample P2. From the loadings plot the greatest activity was associated with compound D which is abundant in samples P1 and P2. As can be seen from Fig B S1 File, the more active samples have a greater abundance of compound D. However, sample P11 is more active than would be predicted from levels of compound D and the activity appears to be based on a combination of the five marker compounds. Compound A seems to be associated with lower activity but not always since it is high in P7 which has relatively high activity. MS^2^ and MS^3^ spectra were obtained for the marker compounds and are described below. The MS^2^ and MS^3^ spectra for these compounds are shown in Figs C-L in S1 File.

**Table 2:** Activity of samples P1-P12 against *P*.*falciparum* (n = 3).

		EC_50_ (μg/mL)			
Compound	1	2	3	Mean	SEM
P1	5.9	6.0	6.2	6.1	0.10
P2	5.3	2.3	2.6	3.4	0.96
P3	7.8	9.6	8.4	8.6	0.52
P4	14.5	13.7	15.4	14.5	0.48
P5	26.8	32.2	27.2	28.7	1.8
P6	40.7	44.1	43.6	42.8	1.0
P7	6.6	13.3	12.1	10.6	2.1
P8	46.7	50.3	63.8	53.6	5.22
P9	7.0	9.8	9.2	8.7	0.84
P10	23.1	20.0	24.9	22.7	1.43
P11	14.9	14.9	14.2	14.7	0.23
P12	14.6	14.9	13.1	14.2	0.57
Chloroquine (nM)	7.4	7.6	7.5	7.5	0.07

**Table 3:** Most important variables determining the activity of P2 in anti-protozoal and anti-microbial tests and important variables determining cellular toxicity based on sample P8 which was the most cytotoxic sample.

[M-H]^-^	Rt (min)	Molecular formula	Compound
*P*.*falciparum*			
373.275	52.6	C_24_H_38_O_3_	Compound A
347.259	52.9	C_22_H_36_O_3_	Compound B
345.244	50.0	C_22_H_34_O_3_	Compound C
341.14	21.4	C_20_H_22_O_5_	Compound D
301.217	43.6	C_20_H_30_O_2_	Compound E
*T*. *brucei*			
373.275	52.6	C_24_H_38_O_3_	Compound A
329.067	13.1	C_17_H_14_O_7_	Compound F
325.145	25.0	C_20_H_22_O_4_	Compound G
301.217	43.6	C_20_H_30_O_2_	Compound E
*L*.*donovani*			
373.275	54.6	C_24_H_38_O_3_	Compound A
325.145	25.0	C_20_H_22_O_4_	Compound D
341.14	13.6	C_20_H_22_O_5_	Compound H
341.103	10.5	C_19_H_18_O_6_	Compound I
*C*. *fasciculata*			
329.067	13.1	C_17_H_14_O_7_	Compound F
325.145	25.0	C_20_H_22_O_4_	Compound G
369.301	47.9	C_22_H_42_O_4_	Compound J
*M*. *marinum*			
341.14	21.4	C_20_H_22_O_5_	Compound D
325.145	25.0	C_20_H_22_O_4_	Compound G
289.108	10.6	C_16_H_18_O_5_	Compound K
369.301	47.9	C_22_H_42_O_4_	Compound J
U937 Cells			
373.275	52.6	C_24_H_38_O_3_	Compound A
341.14	21.4	C_20_H_22_O_5_	Compound D
325.145	25.0	C_20_H_22_O_4_	Compound G
397.275	50.8	C_26_H_38_O_3_	Compound L

**Compound A** C_24_H_38_O_3_, 45 isomers in DNP.

MS^2^ m/z 329.2850 (100) (C_23_H_37_O). MS^3^ (329.2850) No fragmentation at the energy used. Not much information can be derived from the mass spectra since the base peak formed in MS^2^ does not fragment.

**Compound B** C_22_H_36_O_3_, 114 isomers in DNP

MS^2^ m/z 303.2689 (100) (C_21_H_35_O). MS^3^ (303.2689) No fragmentation. Not much information can be derived from the mass spectra since the base peak formed in MS^2^ does not fragment.

**Compound C** C_22_H_34_O_3_, 127 isomers in DNP

MS^2^ m/z 301.2550 (100) (C_21_H_33_O). MS^3^ (301.2550) No fragmentation. Not much information can be derived from the mass spectra since the base peak formed in MS^2^ does not fragment.

**Compound D** C_20_H_22_O_5_, 189 isomers in DNP

MS^2^ 323.1284 (100) (C_20_H_19_O_4_) 313.1287 (C_19_H_21_O_4_) 311.1287 (C_19_H_19_O_4_) 242.0584 (C_14_H_10_O_4_)

MS^3^ (311.1287) 216.0429 (C_12_H_8_O_4_) 188.0479 (C_11_H_8_O_3_) 144.0581 (C_10_H_8_O)

The ion at m/z 144.0581 is an important diagnostic fragment since it corresponds to naphthol and the ion at 188.0479 corresponds to a hydroxylated naphthoic acid. The ion at m/z 216.0429 has an additional CO suggesting a carbonyl is also substituted onto a hydroxynaphthoic acid and this fragment would arise from the molecular ion via the loss of a hydroxylated C_8_H_13_ hydrocarbon chain. It was not possible to correlate this information to any structure in the literature.

**Compound E** C_20_H_30_O_2_, 598 isomers in DNP

MS^2^ 220.1470 (100) (C_14_H_20_O_2_), 205.1235 (C_13_H_17_O_2_)

MS^3^ (220.1470) 205.1235 (100) (C_13_H_17_O_2_)

Not much structural information is revealed from the fragments produced.

#### Activity of propolis extracts against *T*. *brucei*

Fig M in S1 File shows an OPLS model based on four compounds correlating strongly with activity against *T*. *brucei* (Table C in S1 File). Two of these were compounds A and E which were also important in the activity against *P*. *falciparum*. Compounds F and G are discussed below.

**Compound F** C_17_H_14_O_7_, 163 isomers in DNP

MS^2^ m/z 314.0660(100) (C_16_H_10_O_4_) m/z 299.0196 (14.3) (C_15_H_7_O_7_)

MS^3^ (299.0196) m/z 271.0246 (100) (C_14_H_7_O_6_) m/z 255.0299 (6.3) (C_14_H_7_O_5_)

The structure could be related to dimethylquercetin which occurs in temperate propolis. However, the diagnostic fragments which usually arise from cleavage of the C ring in flavonoids were not identified [26].

**Compound G** C_20_H_22_O_4_, 109 isomers in the DNP

MS^2^ m/z 242.0584 (6.1) (C_14_H_10_O_4_) m/z 216.0427 (44.8) (C_12_H_8_O_4_) m/z 188.0477 (65.4) (C_11_H_8_O_3_) m/z 144.0581 (5) (C_10_H_8_O)

MS^3^ (188.0477) m/z 144.0581 (100) (C_10_H_8_O)

This compound is related to compound D but lacks the hydroxyl group in the side chain and thus appears to be a substituted hydroxy naphthoic acid.

#### Activity of propolis extracts against *L*. *donovani*

Only 9 out of 12 propolis samples could be fitted into and OPLS model (Fig N and table D in S1 File). Compounds A and D were important to the model and two additional compounds H and I were also important and are discussed below.

**Compound H** C_20_H_22_O_5_, 189 isomers in DNP

MS^2^ m/z 271.0973 (100) (C_16_H_15_O_4_) m/z 242.0584 (12.0) (C_14_H_10_O_4_) m/z 216.0429 (10.8) (C_12_H_8_O_4_) m/z 188.0479 (14.2) (C_11_H_8_O_3_) m/z 144.0581 (0.8) (C_10_H_8_O)

MS^3^ (271.0973) 242.0584 (100) (C_14_H_10_O_4_) 216.0429 (30.0) (C_12_H_8_O_4_) 188.0479 (46.0) (C_11_H_8_O_3_) 144.0581 (1.8) (C_10_H_8_O)

Compound H is an isomer of compound D and has very similar mass spectrum, and thus is clearly structurally related to compound D.

**Compound I** C_19_H_18_O_6_ Isomers in DNP 128

MS^2^ m/z 323.0923 (19.6) (C_19_H_15_O_5_) m/z 311.0921 (52.8) (C_14_H_10_O_4_) m/z 293.0818 (36.4) (C_18_H_13_O_4_) m/z 265.0479 (10.7) (C_17_H_13_O_3_) m/z 176.0478 (84.2) (C_10_H_8_O_3_)

MS^3^ (m/z 176.0478) m/z 147.0452 (100) (C_9_H_7_O_2_)

Compound I is most probably closely related to the lignan sesamin previously characterised in Libyan propolis [12] but lacks one of the methylene groups, having a catechol structure in one of the aromatic rings rather than a methylene dioxy group.

#### Activity of propolis extracts against *C*. *fasciculata*

The activity against *C*. *fasciculata* (Table E in S1 File) correlated strongly with three compounds in an OPLS model (Fig O in S1 File). Compounds F and G, which were important in other models of activity, also correlated with high activity; compound J correlated with low activity. Compound J is a relatively minor peak and did not afford a clear MS^2^ spectrum.

#### Activity of propolis against *M*. *marinum*

The samples were tested against *M*.*marinum* in order to determine whether or not any observed activity against a mycobacterium might associate with different components in the samples. *M*.*marinum* is of interest since is genetically the closest mycobacterium to *Mycobacterium tuberculosis* [27]. An OPLS model based on four components (Fig P in S1 File) gave a good fit to the activity against *M*. *marinum* (Table F in S1 File). Again compounds D and G were responsible for high activity while compounds J and K correlated with low activity.

#### Toxicity of propolis against mammalian cells (U937)

The toxicity of the propolis extracts was tested against mammalian cells (Table G in S1 File). For three of the samples, P9, P11, P12 there was no significant toxicity up to 100 μg/ml and thus they were excluded from the OPLS model (Fig Q in S1 File). The most toxic sample was P8 which gave an IC_50_ value of 34.1 μg/ml. Of the samples showing toxicity below 100 μg/ml P2 was the least toxic. The main compounds responsible for the toxicity of the samples were compound A and compound L. From the similar elemental compositions it seemed possible that compound A and compound L might be related. The mass spectrum of compound L is discussed below.

**Compound L** C_26_H_38_O_3_, 23 isomers in DNP.

MS^2^ m/z 353.2867 (100) (C_25_H_37_O). MS^3^ (m/z 353.2867) 351.2715 (100) (C_25_H_35_O) m/z 337.2557 (15.7) (C_24_H_33_O_3_), m/z 323.2400 (2.9) (C_23_H_31_O), m/z 309.2243 (5.9) (C_22_H_29_O), m/z 295.2084 (7.3) (C_21_H_27_O), m/z 281.1929 (6.3) (C_20_H_25_O), m/z 267.1771 (5.9) (C_19_H_23_O), m/z 253.1613 (5.6) (C_18_H_21_O), m/z 239.1451 (5.5) (C_17_H_19_O), m/z 225.1299 (3.4) (C_16_H_17_O) m/z 133.0667 (0.8) (C_9_H_9_O), 119.0511 (2.3) (C_8_H_7_O), 107.0509 (2.2) (C_7_H_7_O).

MS^3^ suggested a phenol substituted with a 17 carbon chain containing four units of unsaturation. The compound also contains a carboxylic acid shown by the loss of CO_2_ in the MS^2^ spectrum. The structure is consistent with an anacardic acid, these compounds are found in cashew oil [28]. On closer examination of the MS^3^ spectrum of compound A it was also observed that very small ions corresponding at m/z 119.0511 and 107.0509 could be observed. Thus it seems likely that compound A is also an anacardic acid with substituted with a 17 carbon chain with two units of unsaturation. Looking at the marker compounds in Table 1 all but one of the top 10 VIPs for sample 8, the most toxic sample, have elemental compositions that would fit anacardic acids substituted with varying alkyl chains. Sample P8 is from the SW of the country from an oasis area with a very dry climate thus there is nothing to suggest that cashew trees might grow in this area, however, pistachio trees (*Pistacia vera*) are cultivated in Libya and these contain anacardic acids [29]. A closely related series of alkylated phenols was recently observed in Cameroonian propolis [30] and were thought to originate from the Anacardiaceae family of plants. Anacardic acids have also been observed in propolis from Oman and Brazil [31, 32]. Anacardic acids have been shown to exhibit cytotoxicity [33] and their high levels in P8 would explain why it is the most cytotoxic sample. The samples from the other oasis area in the SE of the country P5/6/7 also contain anacardic acids and are relatively cytotoxic.

## Concluding Remarks

Evidence is mounting that propolis protects bee hives against microbial infection [6, 7, 34–37]. With the problems of colony collapse affecting bee hives in many parts of the world a better understanding of propolis is of great importance. The chemical composition of propolis could potentially reveal a great deal about the interaction between the bee and its environment. What is not known is whether or not bees through selection pressure have targeted plants producing resins with the desired biological properties or it just happens that the plant resins which are suitable the coating of hives just happen to have antimicrobial properties. Strong anti-microbial properties are not universal and in a survey of anti-bacterial activity of propolis from various parts of the world it was found that many samples from Sub-Saharan Africa did not have anti-bacterial properties [38] against the eight types of bacteria studied. In the current case the samples from the South of Libya were less active against protozoa but did exhibit more cytotoxicity. Is this variation just random because the plant sources are varied or is it that the bees face different environmental pressures in different regions? Considering protozoa specifically it is known that these infect insects [8] and it is also known that trypanosomatids occur in plant latexes and in fruits [39]. Thus plants also have an interest in defence against infection against protozoa and it might be expected that some plant resins would have anti-protozoal properties but obviously not all as judged from the current survey. Again a question which arises regarding whether or not plants from certain environments are more likely to face pressure from protozoal infection? The same might be true of bacterial infection and we concluded in our earlier study that propolis from tropical areas with high rain fall and warm temperatures has the highest anti-microbial activity [38]. Thus since nature is so interconnected it might be that bees for instance in an environment where plants do not face pressure from protozoal attack also are not susceptible to this pressure. Protozoal infection might not occur in the dry areas in the South of Libya. However, propolis is still collected by bees in these areas and this might simply be for its properties as a mechanical barrier rather to ward off infection. The propolis from the South of Libya is more cytotoxic and from the plant’s point of view this might be simply to make it unpalatable to animals. Finally there is little doubt the discovery of new anti-protozoal compounds is particularly important. There has been little development of new anti-protozoal drugs for many decades, resistance to the existing treatments has become a problem and the treatments that are used are quite toxic and often have poor bioavailability and have to be given by injection [40, 41]. Although there is a resistance to the notion of using extracts as treatments bees appear to exert a degree of quality control as judged similar activity for samples P1 and P2. Thus could propolis extracts have a role in treating these diseases at low cost and in the process encourage bee keeping?

## Supporting Information

S1 File Fig A PCA separation of propolis samples according to positive ion MS data.
Fig B Abundance of compound D according to chromatographic peak area in the 12 Libyan propolis samples.
**Fig C Compound A** MS^2^ and MS^3^ spectra obtained with a collision energy of 35V.
**Fig D Compound B** MS^2^ and MS^3^ spectra obtained with a collision energy of 35V.
**Fig E Compound C** MS^2^ and MS^3^ spectra obtained with a collision energy of 35V.
**Fig F Compound D** MS^2^ and MS^3^ spectra obtained with a collision energy of 35V.
**Fig G Compound E** MS^2^ and MS^3^ spectra obtained with a collision energy of 35V.
**Fig H Compound F** MS^2^ and MS^3^ spectra obtained with a collision energy of 35V.
**Fig I Compound G** MS^2^ and MS^3^ spectra obtained with a collision energy of 35V.
**Fig J Compound H** MS^2^ and MS^3^ spectra obtained with a collision energy of 35V.
**Fig K Compound I** MS^2^ and MS^3^ spectra obtained with a collision energy of 35V.
**Fig L Compound L** MS^2^ and MS^3^ spectra obtained with a collision energy of 35V.
Fig M OPLS model of the activity of Libyan propolis samples against *T*.*brucei* based on four compounds. P3 was omitted in order to improve the fit of the model.
Fig N OPLS plot of observed against predicted activity of propolis samples against *L*.*donovani*. Samples P3, P6 and P11 were omitted in order to improve the fit of the model.
Fig O OPLS plot of observed against predicted activity of propolis samples against *C*. *fasciculata*. Sample P3 was omitted in order to improve the fit of the model.
Fig P OPLS plot of observed against predicted activity of propolis samples against *M*.*marinum*.
Fig Q OPLS plot of observed against predicted activity of propolis samples against cells. Samples P3 and P12 were omitted in order to improve the fit of the model.
Table A Main plants visited by bees in Libya and their flowering period
Table B The physical properties of the Libyan propolis samples.
Table C Anti-trypanosomal activity of samples P1-P12 against *T*.*brucei* (s427) (n = 3).
Table D IC values obtained for P1-12 against *L*. *donovani* amastigotes (n = 3).
Table E EC_50_ and EC_90_ values μg/ml (n = 4) obtained for propolis extracts against *C*. *fasciculata*.
Table F MIC values for P1-P12 tested against against *M*. *marinum* (n = 2, values identical for the replicates).
Table G Cytotoxicity for P1-9 and P11 measured against U937 cells.
(DOCX)
