# Supplementary material for: Chemical and Antimicrobial Profiling of Propolis from Different Regions within Libya
Source: PLoS One. 2016 May 19;11(5):e0155355. doi: 10.1371/journal.pone.0155355 (PMC4873177; doi:10.1371/journal.pone.0155355)
Supplement: S1 File — Fig A PCA separation of propolis samples according to positive ion MS data. Fig B Abundance of compound D according to chromatographic peak area in the 12 Libyan propolis samples. Fig C Compound A MS2 and MS3 spectra obtained with a collision energy of 35V. Fig D Compound B MS2 and MS3 spectra obtained with a collision energy of 35V. Fig E Compound C MS2 and MS3 spectra obtained with a collision energy of 35V. Fig F Compound D MS2 and MS3 spectra obtained with a collision energy of 35V. Fig G Compound E MS2 and MS3 spectra obtained with a collision energy of 35V. Fig H Compound F MS2 and MS3 spectra obtained with a collision energy of 35V. Fig I Compound G MS2 and MS3 spectra obtained with a collision energy of 35V. Fig J Compound H MS2 and MS3 spectra obtained with a collision energy of 35V. Fig K Compound I MS2 and MS3 spectra obtained with a collision energy of 35V. Fig L Compound L MS2 and MS3 spectra obtained with a collision energy of 35V. Fig M OPLS model of the activity of Libyan propolis samples against T.brucei based on four compounds. P3 was omitted in order to improve the fit of the model. Fig N OPLS plot of observed against predicted activity of propolis samples against L.donovani. Samples P3, P6 and P11 were omitted in order to improve the fit of the model. Fig O OPLS plot of observed against predicted activity of propolis samples against C. fasciculata. Sample P3 was omitted in order to improve the fit of the model. Fig P OPLS plot of observed against predicted activity of propolis samples against M.marinum. Fig Q OPLS plot of observed against predicted activity of propolis samples against cells. Samples P3 and P12 were omitted in order to improve the fit of the model. Table A Main plants visited by bees in Libya and their flowering period Table B The physical properties of the Libyan propolis samples. Table C Anti-trypanosomal activity of samples P1-P12 against T.brucei (s427) (n = 3). Table D IC values obtained for P1-12 against L. donovani amastigo [file pone.0155355.s001.docx]

**Supporting Information**

**Figure A** PCA separation of propolis samples according to positive ion MS data.

**
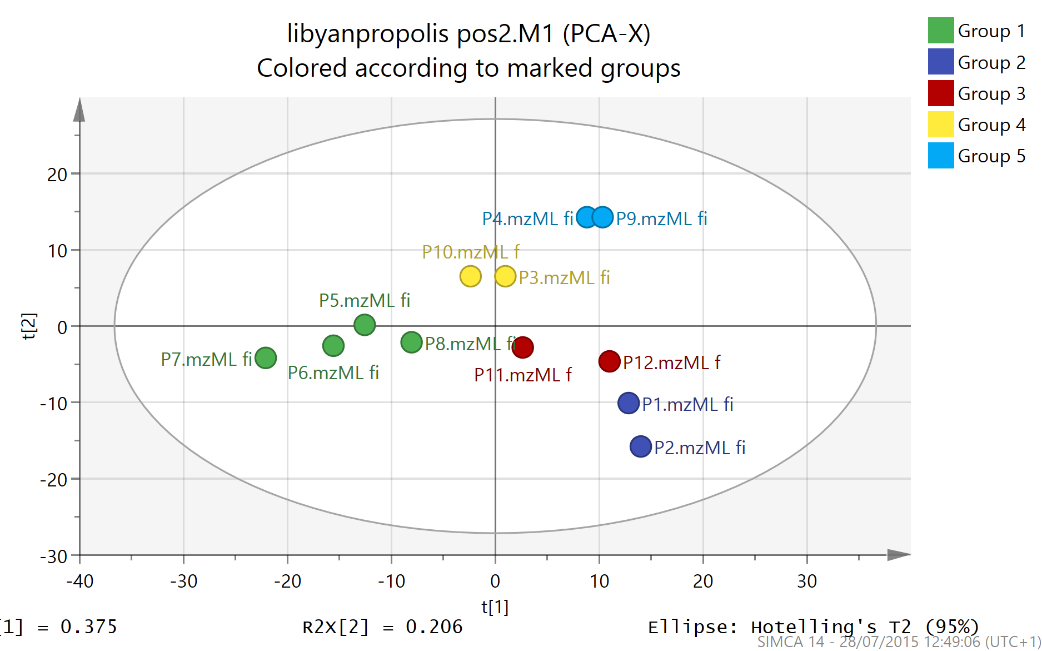
**

**Figure B** Abundance of compound D according to chromatographic peak area in the 12 Libyan propolis samples.

**Figure C Compound A** MS^2^ and MS^3^ spectra obtained with a collision energy of 35V.

**Figure D Compound B** MS^2^ and MS^3^ spectra obtained with a collision energy of 35V.

**Figure E Compound C** MS^2^ and MS^3^ spectra obtained with a collision energy of 35V.

**Figure F Compound D** MS^2^ and MS^3^ spectra obtained with a collision energy of 35V.

**Figure G Compound E** MS^2^ and MS^3^ spectra obtained with a collision energy of 35V.

**Figure H Compound F** MS^2^ and MS^3^ spectra obtained with a collision energy of 35V.

**Figure I Compound G** MS^2^ and MS^3^ spectra obtained with a collision energy of 35V.

**Figure J Compound H** MS^2^ and MS^3^ spectra obtained with a collision energy of 35V.

**Figure K Compound I** MS^2^ and MS^3^ spectra obtained with a collision energy of 35V.

**Figure L Compound L** MS^2^ and MS^3^ spectra obtained with a collision energy of 35V.

**Figure M** OPLS model of the activity of Libyan propolis samples against *T.brucei* based on four compounds. P3 was omitted in order to improve the fit of the model.


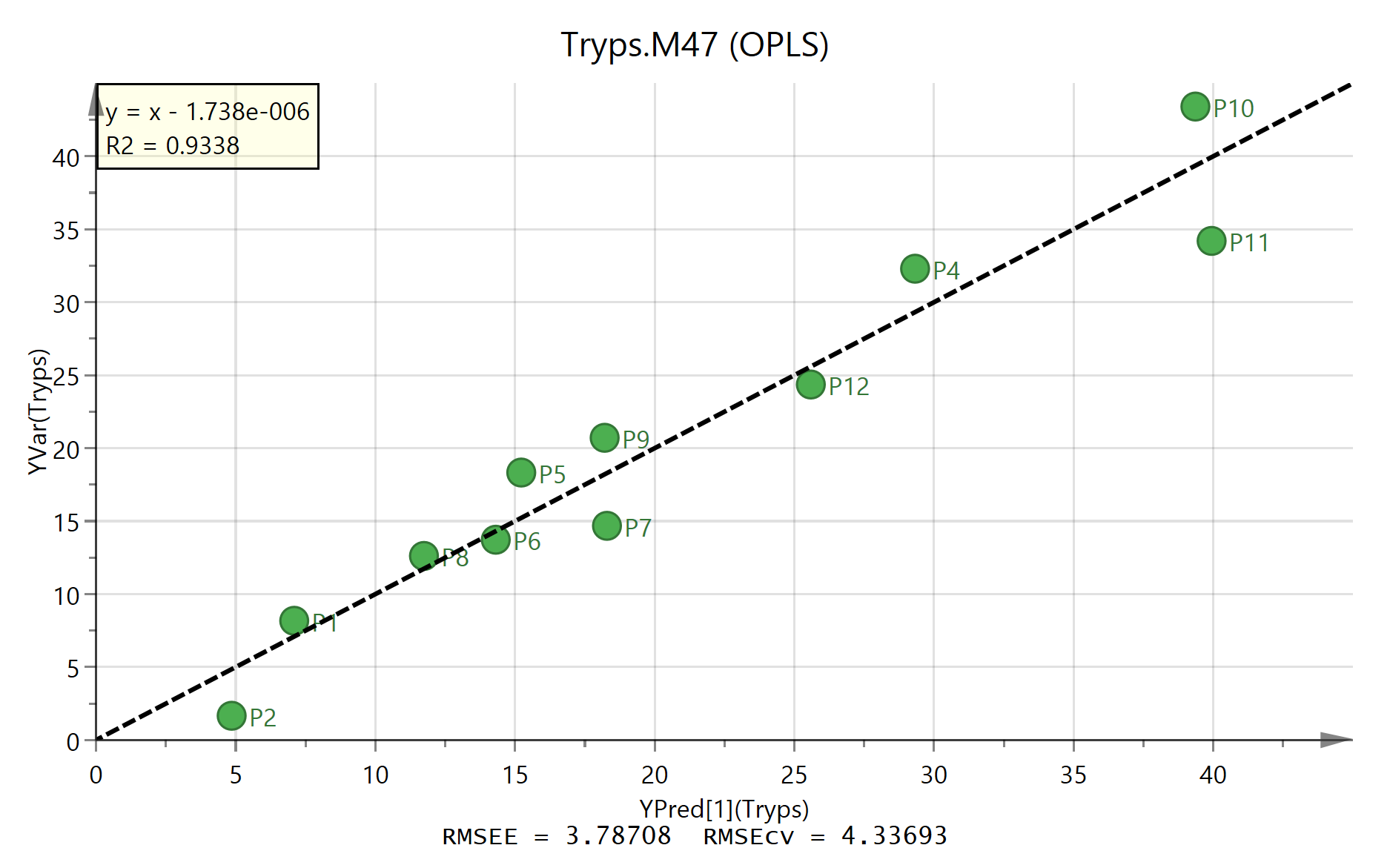


**Figure N** OPLS plot of observed against predicted activity of propolis samples against *L.donovani*. Samples P3, P6 and P11 were omitted in order to improve the fit of the model.


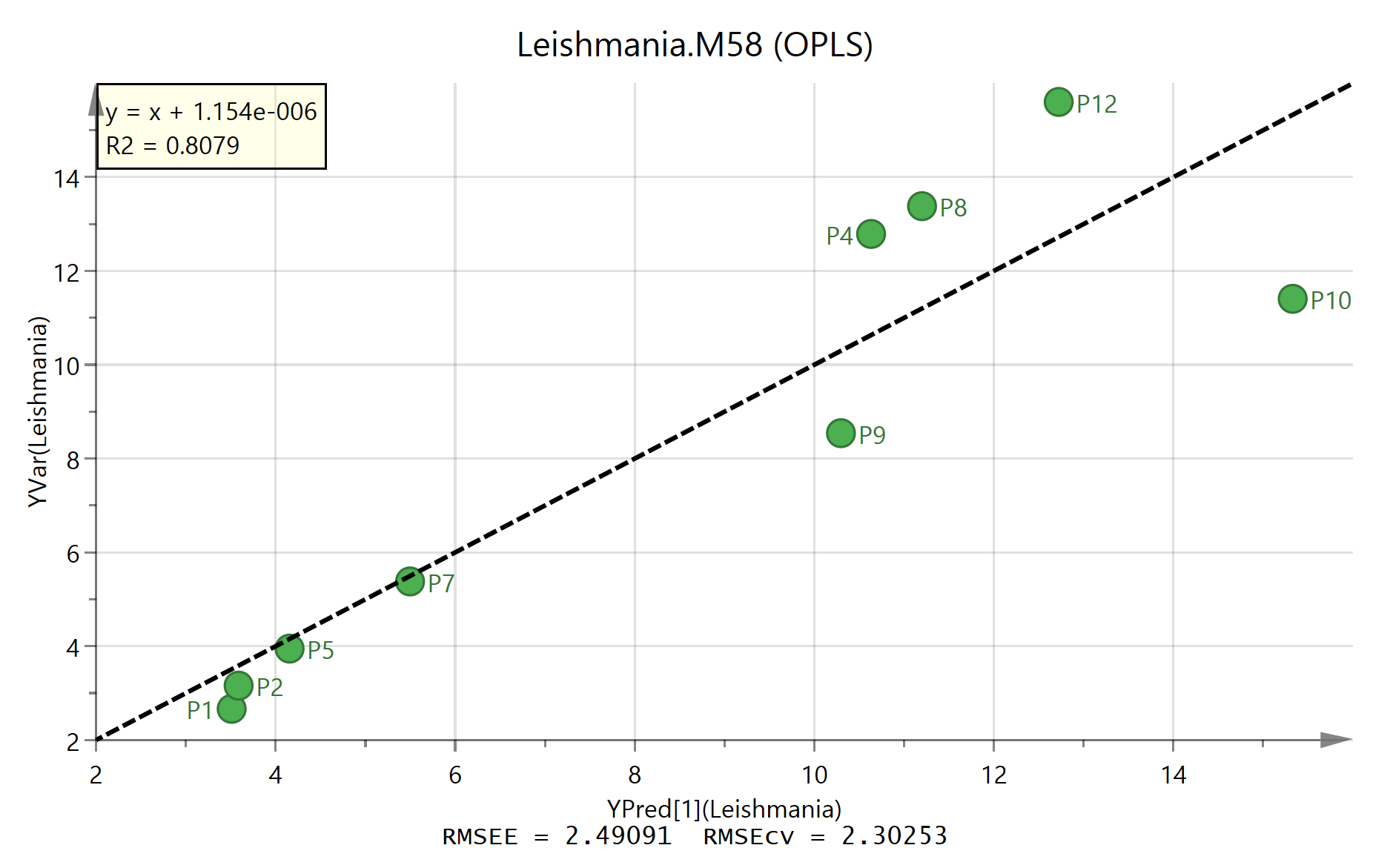


**Figure O** OPLS plot of observed against predicted activity of propolis samples against *C. fasciculata*. Sample P3 was omitted in order to improve the fit of the model.


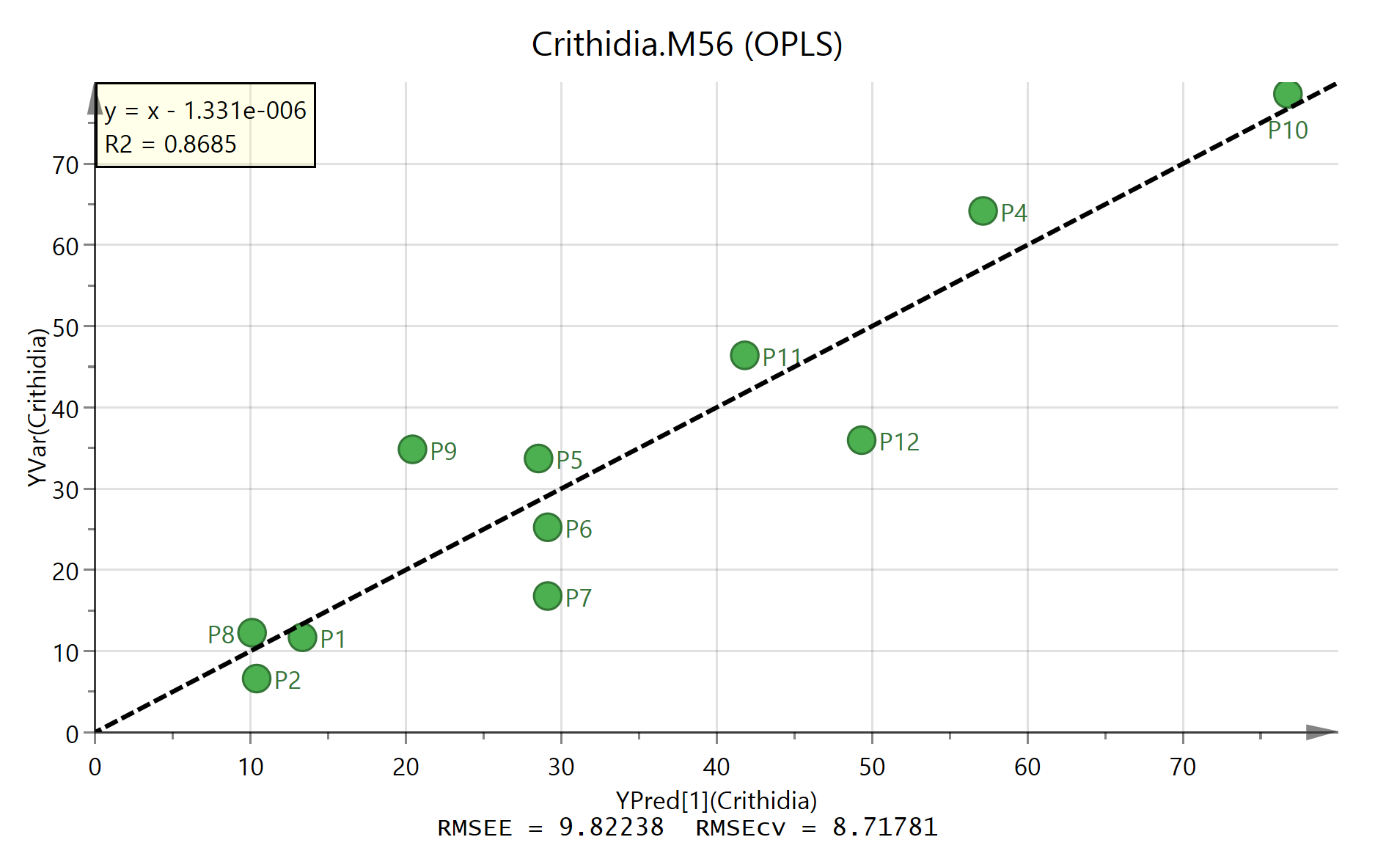


**Figure P** OPLS plot of observed against predicted activity of propolis samples against *M.marinum*.


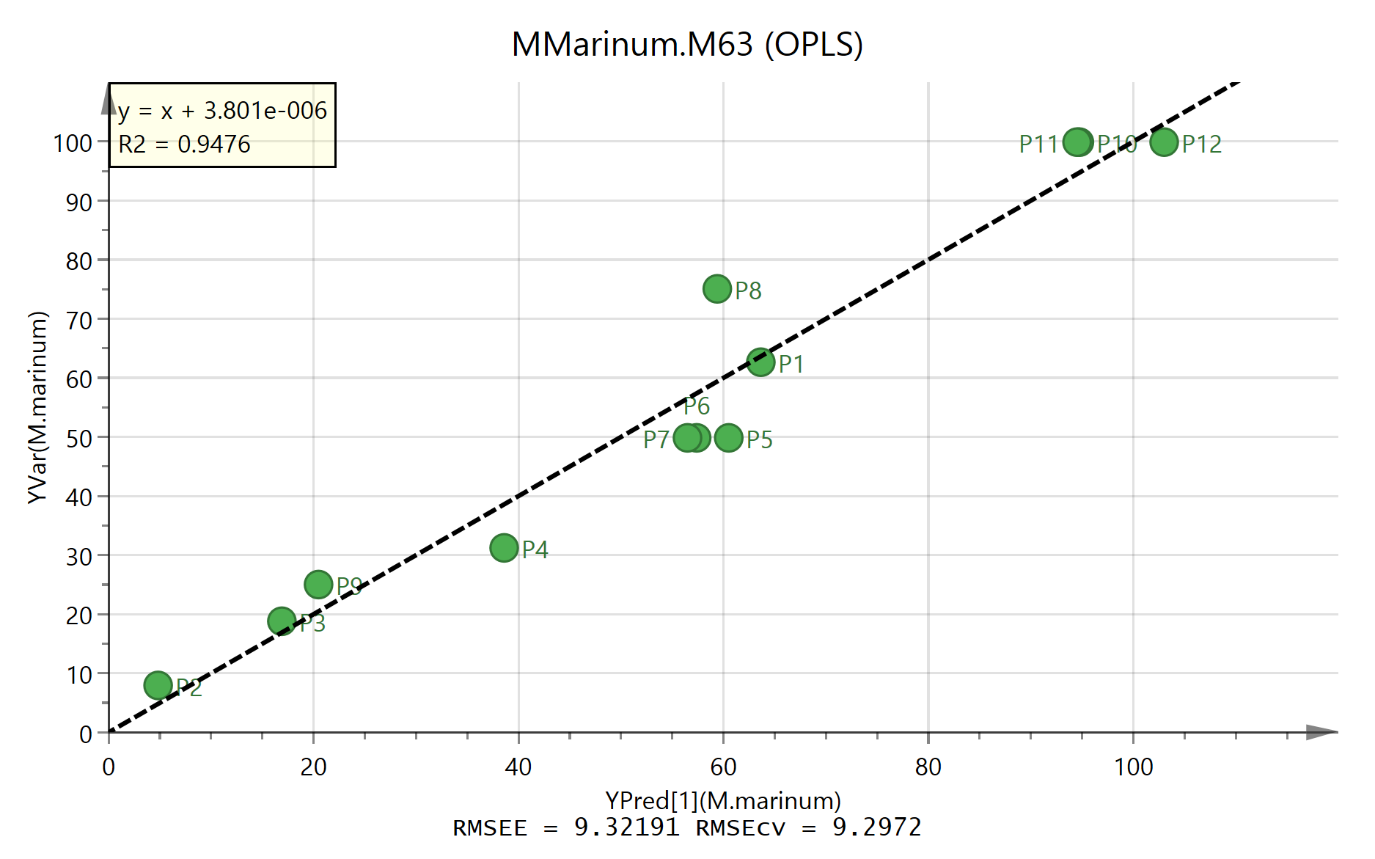


**Figure Q** OPLS plot of observed against predicted activity of propolis samples against U937 cells. Samples P3 and P12 were omitted in order to improve the fit of the model.


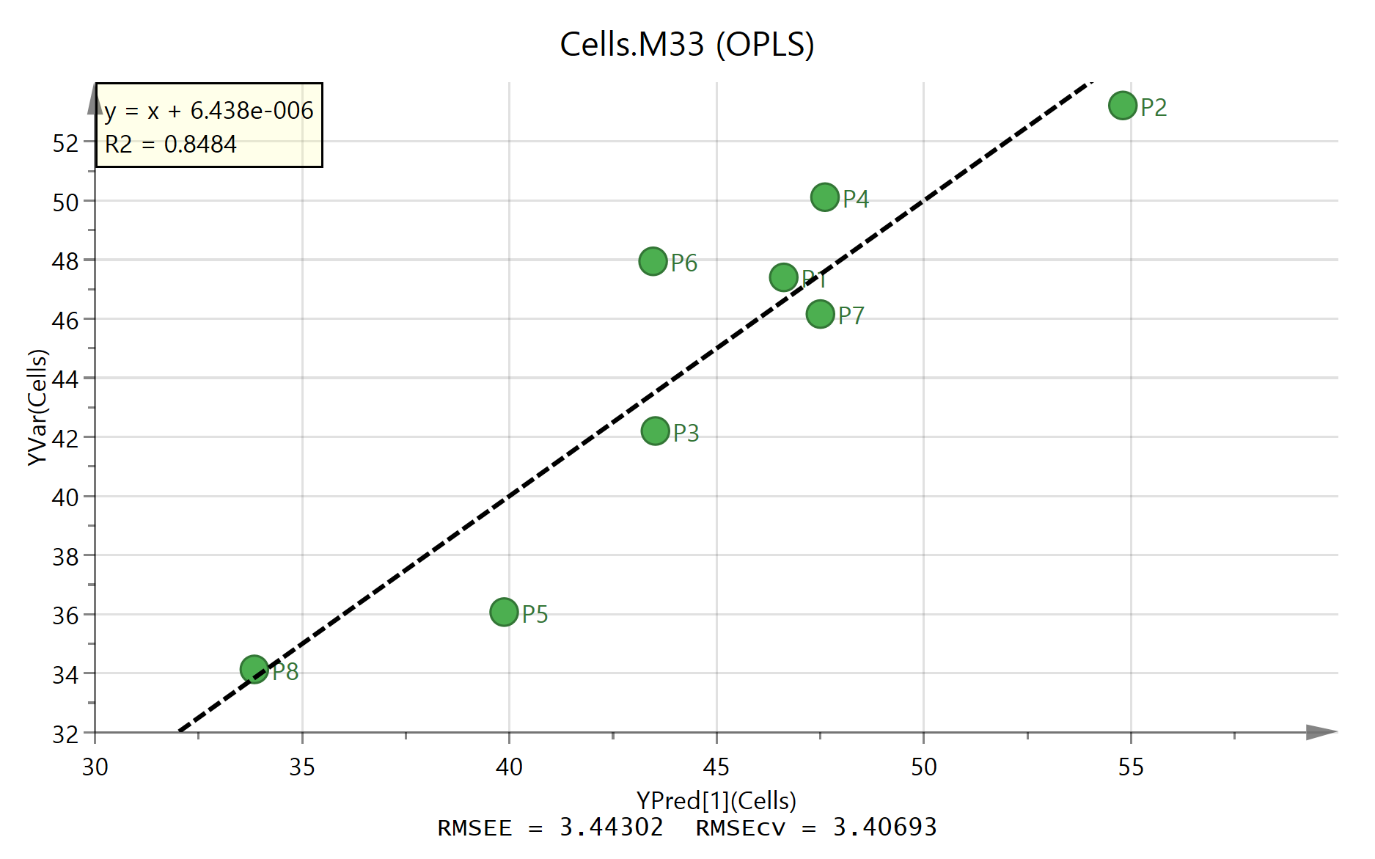


**Table A** Main plants visited by bees in Libya and their flowering period

| **Common name** | **Scientific name** | **Flowering season** |
| --- | --- | --- |
| Orange tree | *Citrus* spp | March – April |
| African rue (harmal) | *Peganum harmala* | April – May |
| Sedr Zizaphus S | *Pina christi* | May – June |
| Thyme (Za’atar) | *Thymus capitatus* L. | June – July |
| Tamarix | *Tamarix africana* | July –August |
| Carob tree | *Ceratonia siliqua* | August-October |
| Schamiry | *Arbutus pavarii* | December – January |

(Adapted from [11])

**Table B** The physical properties of the Libyan propolis samples.

| **Propolis Sample code** | **Total sample weight(gm)** | **Extracted weight (gm)** | **Colour** | | **Odour** | | **Consistency** | |
| --- | --- | --- | --- | --- | --- | --- | --- | --- |
| P1 | 23.04 | 10.35 | Dark brown | | Orange odour | | Sticky | |
| P2 | 20.21 | 8.34 | Dark red brown | | Intense odour | | Semi sticky | |
| P3 | 19.83 | 6.48 | Light brown | | Olive leaf odour | | non sticky | |
| P4 | 20.21 | 6.95 | Dark yellow | | Mild | | Sticky | |
| P5 | 25.04 | 7.45 | Dark brown | | No odour | | Very sticky | |
| P6 | 25.07 | 6.95 | Dark brown | | No odour | | Very sticky | |
| P7 | 41.94 | 20.00 | Dark brown | | Mild | | Very sticky | |
| P8 | 5.80 | 2.35 | Yellow | | Flowery | | Non-sticky | |
| P9 | 13.85 | 5.96 | Dark yellow –light honey | | Intense orange –flower | | Semi sticky | |
| P10 | 15.44 | 4.36 | Light brown | Flowery | | non sticky | |  |
| P11 | 20.47 | 5.41 | Light brown | | Mild odour | | Sticky | |
| P12 | 20.54 | 6.41 | Honey –brown | | Mild odour | | Sticky | |

**Table C** Anti-trypanosomal activity of samples P1-P12 against *T.brucei* (s427) (n=3).

|  |  |  |  |  |  |
| --- | --- | --- | --- | --- | --- |
| EC50 (µg/ml) | | | | | |
|  |  |  |  |  |  |
| TEST COMPOUNDS | EXP. 1 | EXP 2 | EXP 3 | AVG | SEM |
| P1 | 8.47 | 8.89 | 7.35 | 8.24 | 0.33 |
| P2 | 1.67 | 1.68 | 1.60 | 1.65 | 0.02 |
| P3 | 13.89 | 12.53 | 14.05 | 13.49 | 0.34 |
| P4 | 33.33 | 34.04 | 29.49 | 32.29 | 1.00 |
| P5 | 18.73 | 18.84 | 17.18 | 18.25 | 0.38 |
| P6 | 13.65 | 13.73 | 13.71 | 13.70 | 0.02 |
| P7 | 15.61 | 14.38 | 14.01 | 14.67 | 0.34 |
| P8 | 13.14 | 12.85 | 11.98 | 12.66 | 0.25 |
| P9 | 22.53 | 22.08 | 17.33 | 20.65 | 1.18 |
| P10 | 39.38 | 45.35 | 45.35 | 43.36 | 1.41 |
| P11 | 34.75 | 29.15 | 38.59 | 34.16 | 1.94 |
| P12 | 24.50 | 23.83 | 24.90 | 24.41 | 0.22 |
| Pentamidine (µM) | 4.40E-03 | 3.74E-03 | 4.07E-03 | 0.0041 | 0.0001 |

**Table D** IC values obtained for P1-12 against *L. donovani* amastigotes (n=3).

| **Compound** | **Mean IC_50_ value ± SD** |
| --- | --- |
| P1 | 2.6 ± 0.18 |
| P2 | 3.2 ± 0.27 |
| P3 | 11.7 ± 1.1 |
| P4 | 13.4 ± 1.3 |
| P5 | 3.5 ± 0.73 |
| P6 | 10.2 ± 0.26 |
| P7 | 5.4 ± 0.06 |
| P8 | 14.0 ± 1.6 |
| P9 | 8.56 ± 0.34 |
| P10 | 11.0 ± 0.60 |
| P11 | 7.2 ± 0.27 |
| P12 | 16.2 ± 1.5 |

**Table E** EC_50_ and EC_90_ values μg/ml (n=4) obtained for propolis extracts against *C. fasciculata*.

| Code P | EC_50_ | SD | EC_90_ | SD | Regrowth after incubation. |
| --- | --- | --- | --- | --- | --- |
| P1 | 11.6 | 2.1 | 58.1 | 3.2 | N |
| P2 | 6.5 | 1.3 | 39.7 | 2.4 | N |
| P3 | 12.2 | 2.6 | 57.3 | 4.4 | N |
| P4 | 64.1 | 5.3 | >200 |  | Y |
| P5 | 33.7 | 2.3 | 115 | 8.2 | Y |
| P6 | 25.3 | 3.2 | 88.7 | 9.4 | Y |
| P7 | 16.7 | 1.1 | 46.8 | 3.2 | N |
| P8 | 12.4 | 1.6 | 39.2 | 4.3 | N |
| P9 | 34.9 | 3.8 | 148 | 9.5 | Y |
| P10 | 78.5 | 8.6 | >200 |  | Y |
| P11 | 46.3 | 5.5 | 126.4 | 11.4 | Y |
| P12 | 36.1 | 3.8 | 81.1 | 7.9 | Y |
| Menadione | 0.8 | 0.2 | 5 | 0.3 | N |
| Pentamidine | 18.3 | 3.8 | 70.6 | 8.5 | N |

**Table F** MIC values for P1-P12 tested against against *M. marinum* (n=2, values identical for the replicates).

| Sample | MIC μg/ml |
| --- | --- |
| P1 | 62.5 |
| P2 | 7.8 |
| P3 | 18.75 |
| P4 | 31.25 |
| P5 | 50 |
| P6 | 50 |
| P7 | 50 |
| P8 | 75 |
| P9 | >100 |
| P11 | >100 |
| Gentamycin | 6.25 |

**Table G** Cytotoxicity for P1-9 and P11 measured against U937 cells.

| Sample | IC_50_ μg/ml |
| --- | --- |
| P1 | 47.5 |
| P2 | 53.2 |
| P3 | 40.2 |
| P4 | 50.08 |
| P5 | 36.09 |
| P6 | 47.92 |
| P7 | 46.17 |
| P8 | 34.1 |
| P9 | >100 |
| P11 | >100 |
| P12 | >100 |
